# Haemostatic, Inflammatory, and Haematological Biomarkers Among Orthopaedic Patients With Prolonged Immobilization and the Risk of Hypercoagulable States

**DOI:** 10.7759/cureus.51552

**Published:** 2024-01-02

**Authors:** Noor Nabila Ramli, Salfarina Iberahim, Noor Haslina Mohd Noor, Zefarina Zulkafli, Tengku Muzaffar Tengku Shihabuddin, Mohd Hadizie Din, Ahmad Hadif Zaidin Samsudin, Marne Abdullah

**Affiliations:** 1 Department of Hematology, Universiti Sains Malaysia School of Medical Sciences, Kota Bharu, MYS; 2 Department of Hematology, Hospital Universiti Sains Malaysia, Kota Bharu, MYS; 3 Department of Orthopedics, Universiti Sains Malaysia School of Medical Sciences, Kota Bharu, MYS; 4 Department of Radiology, Universiti Sains Malaysia School of Medical Sciences, Kota Bharu, MYS

**Keywords:** venous thromboembolism, haematological biomarker, haemostatic biomarker, inflammatory biomarker, prolonged immobilization, trauma and orthopaedic

## Abstract

Background

Venous thromboembolism (VTE) is a common and potentially life-threatening complication in patients with lower limb traumatic fractures. Orthopaedic patients who experience trauma in the lower limbs with prolonged immobilization may experience a hypercoagulable state, which could eventually lead to the development of VTE. Therefore, this study aims to evaluate the changes in hypercoagulable markers, including haemostatic, inflammatory, and haematological biomarkers in orthopaedic trauma patients with prolonged immobilization.

Materials/method

This prospective cohort study was conducted at Hospital Universiti Sains Malaysia from August 2020 to March 2022. Every patient with fractures in the lower limbs was screened for eligibility, and patients who required immobilization for more than five days without receiving anticoagulant prophylaxis were recruited for this study. The laboratory tests, including D-dimer, fibrinogen, prothrombin time (PT), activated partial thromboplastin time (aPTT), erythrocyte sedimentation rate (ESR), and platelet count, were serially measured on day one and day five of hospitalization. The biomarkers were analyzed using a paired t-test, with a p-value <0.05 as a significant result.

Results

A total of 54 patients with fractures in the lower limbs, ages ranging from 12 to 50 years old, were involved in this study. The paired t-test analysis demonstrated that several biomarkers showed a significant increase in mean difference between day one and day five of immobilization, which included fibrinogen, ESR, and platelet count. The mean differences for each biomarker with fibrinogen were 0.66 g/L (p<0.001, 95% CI of mean difference: -1.04, -0.27), ESR increased by 17.98mm/hr (p<0.001, 95% CI of mean difference: -24.69, -11.27), and platelet count increased by 128.59×10^9^/L (p<0.001, 95% CI of mean difference: -166.55, -90.64) on day five of immobilization. D-dimer was elevated in all patients on both post-trauma days; however, no significant difference was observed in this biomarker between day one and day five of immobilization.

Conclusion

In conclusion, our study found that fibrinogen, ESR, and platelet count levels were significantly increased in orthopaedic trauma patients with prolonged immobilization. The increase in these biomarkers indicates the body's reaction to tissue injury after trauma, which may contribute to the hypercoagulable states. Further research with a larger sample size is warranted to assess the viability of these biomarkers as potential diagnostic indicators for the development of VTE related to hypercoagulability.

## Introduction

Deep vein thrombosis (DVT) and pulmonary embolism (PE) are collectively known as venous thromboembolism (VTE). VTE affects millions of people globally and is one of the primary causes of morbidity and mortality among inpatients [1]. A study reported an incidence rate of VTE ranging from 5% to 63% in trauma patients not receiving prophylaxis [2]. Apart from trauma, temporary immobilization of the lower limb also contributes to the risk of VTE. According to the Cochrane review published by Zee et al., the incidence rate of VTE ranged from 4% to 40% in patients with lower limb immobilization [3].

The combination of orthopaedic trauma and prolonged immobilization increases the risk of VTE. A study found that individuals who were immobilized as a result of trauma exhibited a greater prevalence of VTE compared with those immobilized without trauma [4]. Trauma per se may induce a hypercoagulable state, which in turn may complicate patient conditions through pathological thrombosis. This was confirmed in a study by Selby et al. in 2009 involving multi-system trauma patients, where they reported the overall VTE rate was 59%. However, the sequential changes in coagulation markers have been poorly characterized [5].

DVT is often diagnosed during emergency room visits, and various studies have been undertaken to analyze its risk factors. Due to the significant morbidity and mortality risks associated with VTE, the prevention, diagnosis, and management of this disease in orthopaedic trauma patients are crucial. The diagnosis of DVT using blood biomarkers is not well established. D-dimer is the only biomarker that has been successfully used in clinical practice as a predictive tool for diagnosing DVT [6]. Several studies found that screening criteria based on D-dimer levels can serve as a reference to rule out VTE in severe trauma patients [7] and postoperative lower limb fracture patients [8]. Nevertheless, another study indicated that D-dimer serves not only as a marker for identifying DVT and PE but also as an indicator for assessing the severity of trauma in patients with acute injuries. In other words, D-dimer alone cannot be utilized as a predictive marker for VTE in trauma patients [9]. Given its limitations, more research into potential additional biomarkers for identifying VTE is required.

Although there is a vast amount of information on VTE in the overall trauma population, the research lacks sufficient data to support firm recommendations regarding the type and duration of prophylaxis that should be given to these patients [10]. The use of pharmacological thromboprophylaxis could minimize the future incidence of VTE in these patients. However, given the lack of knowledge regarding the actual VTE risk and the scarce evidence on risk stratification, there is considerable diversity in the global practices of thromboprophylaxis [11]. Therefore, this study aims to evaluate the changes in hypercoagulable markers (haemostatic, inflammatory, and haematological parameters) in patients with prolonged immobilization due to trauma.

## Materials and methods

Study design and sample selection

This prospective cohort study among orthopaedic trauma patients with prolonged immobilization was conducted at Hospital Universiti Sains Malaysia from August 2020 to March 2022. The inclusion criteria included all patients with prolonged immobilization due to fractures involving the lower limbs (post-trauma), aged between 12 and 50 years, and patients who were confined to bed for more than five days post-trauma with traction and not on anticoagulant prophylaxis. Immobilization in this study is defined as the restriction of a limb or patient movements to allow healing; to reduce or eliminate motion of (the body or a specific part) by mechanical means, whether by above or below knee cast or by strict bed rest (confined to the bed with traction). The selection of day five as the endpoint for prolonged immobilization was guided by a combination of clinical relevance and practical considerations. Based on existing literature, it is well established that being bedridden or immobilized for more than three days poses a higher risk of VTE [12]. A meta-analysis conducted by Testroote et al. revealed a range of DVT incidences, varying from 4.3% to 40% among immobilized patients with a minimum duration of one week without prophylactic measures [13]. In our study, the selection of day five for the analysis of the second sample corresponds to the circumstance wherein the majority of patients in our clinic had been encouraged to resume mobility. In addition, patients in this study were not on anticoagulant prophylaxis due to their low risk of VTE.

The exclusion criteria included patients with known cases of thrombophilia and anti-phospholipid syndrome (APS); patients on prophylactic anticoagulant and antiplatelet therapy; patients with a history of VTE or symptomatic patients requiring anticoagulant during the study or at presentation; past medical history of ischaemic heart disease (IHD) or diabetes mellitus (DM); females on oral contraceptive pills (OCP) or pregnant; and disseminated intravascular coagulation (DIC), liver disease, sepsis, or complicated fractures involving the spine, pelvis, and brain injuries. Ethical approval for the study was given by the Human Research Ethics Committee of Universiti Sains Malaysia on July 2, 2020 (USM/JEPeM/19120979).

Blood collection

Eligible patients were recruited, and written informed consent was obtained on day one of admission. The clinical risk factors recorded were gender, age, type of injury, body mass index (BMI), and smoking status. Patient blood was collected two times serially on day one of admission (within 24 hours of injury) and day five of immobilization. The blood sample was tested for D-dimer, fibrinogen, prothrombin time (PT), activated partial thromboplastin time (aPTT), erythrocyte sedimentation rate (ESR), and platelet count. The samples were collected into one ethylenediaminetetraacetic acid (EDTA) bottle (3 mL) and one trisodium citrate bottle (2.7 mL). The samples from citrated bottles were centrifuged for 15 minutes at 2500g at room temperature to obtain platelet-poor plasma (PPP) and were immediately tested for D-dimer, fibrinogen, PT, and aPTT. These samples were run within two to four hours from the time of venepuncture. The samples from the EDTA tube were analyzed immediately for a full blood count (FBC) to get platelet count and ESR. The FBC was done by automation, and ESR was done by semi-automation.

A coagulation profile that included D-dimer, fibrinogen, PT, and aPTT was performed using an automated analyzer, STA-R Max (Stago: USA). Platelet count was performed on automation, the XN-1000 (Sysmex: Japan), while ESR was performed on a semi-automated analyzer, Mixrate-X20 analyzer (ELITech Group: Netherlands).

Statistical analysis

Data were recorded and analyzed using the IBM SPSS Statistics for Windows, Version 26 (Released 2019; IBM Corp., Armonk, New York). Descriptive statistics were used to summarize the clinical characteristics of the patient sample. Statistical comparison was done by paired t-test to determine the mean difference in haematological, inflammatory, and haemostatic biomarker changes between day one and day five of immobilization. A p-value of less than 0.05 was considered statistically significant at a confidence level of 95%.

## Results

A total of 54 patients with lower limb/s fractures who required immobilization for more than five days and received no anticoagulant prophylaxis were involved in this study. Most of the patients were males, contributing approximately 90.7% (n=49) of the patients, and only 9.3% (n=5) were females. The age distribution ranged from 12 to 50 years, with the majority of patients coming from the 12-20 and 21-30 age groups, contributing around 37.0% (n=20) in each group. Meanwhile, the age distribution for patients in the age groups of 31-40 and 41-50 years was about 16.7% (n=9) and 9.3% (n=5), respectively. The majority of patients involved in this study had a single lower limb injury, accounting for around 59.3% (n=32), compared to multiple injuries at around 40.7% (n=22). Approximately 35.2% (n=19) of the patients in this research were overweight, and 55.6% (n=30) were smokers. Additional details about the patient characteristics are presented in Table 1.

**Table 1:** Characteristics of patients (n=54)

Characteristic	Overall	
	n	%
Gender		
Male	49	90.7
Female	5	9.3
Age		
12–20	20	37.0
21–30	20	37.0
31–40	9	16.7
41–50	5	9.3
Type of injury		
Single lower limb	32	59.3
Multiple injuries	22	40.7
BMI (kg/m^2^)		
Less than 18.4	12	22.2
18.5–24.9	23	42.6
25.0–29.9	19	35.2
Smoke		
Smoking	30	55.6
Non-smoking	24	44.4

Among the haemostatic, inflammatory, and haematological parameters studied, only fibrinogen, ESR, and platelet count showed significant mean differences between day one and day five of immobilization (Table 2). The mean fibrinogen level was higher on day five compared to day one, with the mean fibrinogen increased by 0.66g/L (p<0.001). The mean ESR increased by 17.98 mm/hour (p<0.001), while the mean platelet count increased by 128.59×10^9^/L (p<0.001) on day five of immobilization. Furthermore, both the PT and D-dimer tests showed a decrease in mean level, while aPTT showed an increase in mean level between day one and day five of immobilization. However, these parameters did not show any significant changes in patients with prolonged immobilization.

**Table 2:** Haemostatic, inflammatory, and haematological changes associated with prolonged immobilization patients ^a^Paired t-test was applied. p<0.05 is taken as significant at a 95% confidence interval.

Variable (reference range)	Day 1, mean ± SD	Day 5, mean ± SD	Mean difference (95% CI)	t-statistic (df)^a^	p-value
D-dimer (<0.50 µg/mL)	2.45 ± 1.91	2.28 ± 1.70	–0.18 (–0.24, 0.59)	–0.84 (53)	0.405
Fibrinogen (2.32–4.44 g/L)	4.20 ± 1.08	4.85 ± 1.43	0.66 (–1.04, –0.27)	3.43 (53)	<0.001
PT (12.61–15.72 s)	14.31 ± 1.11	14.10 ± 1.35	–0.21 (–0.04, 0.47)	–1.69 (53)	0.098
aPTT (30.00–45.80 s)	39.48 ± 6.28	40.48 ± 6.34	1.00 (–2.16, 0.16)	1.73 (53)	0.090
ESR (1.0–20.0 mm/hr)	19.81 ± 17.17	37.80 ± 32.01	17.98 (–24.69, –11.27)	5.37 (53)	<0.001
Platelet count (150-400×10^9^/L)	246.04 ± 67.34	374.63 ± 157.59	128.59 (–166.55, –90.64)	6.80 (53)	<0.001

## Discussion

This is a prospective cohort study in post-lower limb trauma patients that evaluates changes in haemostatic, inflammatory, and haematological parameters in response to prolonged immobilization. This study assessed several laboratory parameters that were potential biomarkers contributing to post-traumatic hypercoagulable states and VTE development. The fact that none of the participants were taking anticoagulants provided a good opportunity to investigate the biomarkers involved. Among all laboratory parameters tested, fibrinogen, ESR, and platelet count were found to have a significant mean difference between day one and day five post-trauma and immobilization. The level was higher on day five following trauma and immobilization. Despite the considerable increase in these biomarkers, none of the patients developed VTE throughout the study period. This indicates that additional concomitant risk factors are required to exert a significant impact on the hypercoagulable state of these biomarkers.

Fibrinogen is known as an acute-phase protein. Trauma or injury triggers the acute phase response, a systemic reaction of the body to inflammation or tissue damage. This response involves the release of various proteins, including fibrinogen, by the liver. In the occurrence of traumatic incidents, there is the potential for these proteins to escalate up to four times beyond their normal levels [14]. Fibrinogen plays a crucial role in contributing to a hypercoagulable state when its levels increase. It is a key component in the blood clotting cascade, and elevated levels can lead to an imbalance in the coagulation system, promoting the formation of excessive blood clots by increasing the velocity of platelet aggregation and platelet reactivity [15]. Lin et al. discovered that an increase in fibrinogen levels following traumatic fracture might contribute to hypercoagulable states and subsequently increase the risk of thrombosis [16]. Similarly, another study discovered that fibrinogen levels increased on day five of trauma, leading to a hypercoagulable state [17]. Several studies have reported that an elevated plasma fibrinogen level is linked to a greater risk of VTE [18-20].

In this study, elevated ESR levels were observed in these trauma patients on day five of immobilization. The findings aligned with a previous study by Yousuf et al., who discovered that ischemic tissue damage or trauma is one of the causes that can significantly increase the levels of ESR [21]. The increase in the ESR value occurs as a result of inflammation. This inflammation leads to an increase in the number of proteins in the blood, such as fibrinogen, prothrombin, CRP, plasminogen, and complement proteins. A high level of these proteins causes red blood cells to clump together and form a stack called rouleaux. The formation of rouleaux allows the red blood cells to settle at a faster rate, thus increasing the ESR [22]. According to a study by Chen et al., it was found that the results for ESR and platelet count in the DVT group were significantly higher than those in the non-DVT group [23].

Platelet counts were noted to significantly increase in these trauma patients when comparing the mean level between day one and day five of immobilization. The increase in platelet count is part of the body's physiological response to trauma. Trauma induces the activation of platelets through various mechanisms, including exposure to damaged endothelial cells and the release of signalling molecules. Activated platelets change shape, become sticky, and adhere to the exposed collagen at the injury site. Additionally, trauma triggers the release of various signalling molecules, such as thrombopoietin, which stimulates the bone marrow to produce more platelets [24]. The findings were supported by Salim et al., who found that 18.7% of post-traumatic patients developed thrombocytosis. They also reported a VTE incident rate of 2.4% in individuals who developed thrombocytosis following trauma, with a higher tendency for DVT and a significantly higher risk of PE (p<0.0032) [25]. Meanwhile, Harr et al. found that platelets were a dominant contributor to a hypercoagulable state post-injury. Based on the in-vitro study, they demonstrated that increased platelet counts caused an increased fibrin production, which in turn caused increased thrombus formation. From these findings, they suggested the important role of considering antiplatelet therapy in VTE prophylaxis following trauma, particularly after 48 hours post-trauma [26].

In this study, a quantitative assay was used for the measurement of D-dimer. D-dimer levels were observed to surpass the normal range (<0.50 μg/mL) on both days for all patients. However, these biomarkers exhibited no statistically significant difference when compared between day one and day five of immobilization. D-dimer is a degradation product of circulating cross-linked fibrin produced following the activation of the fibrinolytic pathway of the coagulation system. It has been widely used in clinical practice to aid in the diagnosis of VTE. Several studies have observed that increases in the D-dimer value were related to a greater risk of VTE [27]. Elevated plasma D-dimer levels are common in trauma patients. Zhang et al., in their study, found that the increase in D-dimer levels is closely related to the number of fractures in trauma patients. Furthermore, they concluded that D-dimer levels were utilized not only as a predictor of DVT and PE but also as an indicator of the severity of trauma in patients suffering from acute trauma [9]. Therefore, our data suggests that relying solely on D-dimer is insufficient for evaluating the risk of VTE associated with hypercoagulability in trauma patients with prolonged immobilization.

Additionally, our study reported a slight reduction in PT levels in trauma patients with prolonged immobilization. However, no significant difference could be seen in PT levels between day one and day five of immobilization. A few underlying mechanisms contribute to this phenomenon. Trauma patients with prolonged immobilization often result in decreased physical activities. The lack of movement during immobilization can lead to venous stasis, particularly in the lower extremities [28]. This venous stasis can contribute to the activation of coagulation factors, leading to a reduction in PT levels [29].

Although the findings of this study showed significant results for these three prothrombotic biomarkers (fibrinogen, ESR, and platelet count), none of the trauma patients with prolonged immobilization developed VTE during the study period. This might be due to the small sample size and selective subject criteria for this study. The healthy and young patients included in this study were expected to have a low risk of developing VTE. Additionally, the patients were followed for a very short period of time (only up to day five) to assess VTE development. A significant number of patients were discharged early, and the majority of patients were urged to mobilize as soon as possible following surgery to lower the risk of VTE. The small sample size decreased the statistical power of the study, potentially allowing significant results to go unnoticed. A more comprehensive multi-centre investigation, including several trauma centres throughout Malaysia, is necessary to resolve this. This approach would allow for a better representation of the actual risk of VTE and the clinical significance of these biomarkers.

## Conclusions

In conclusion, we found three biomarkers that showed significant changes after day five of immobilization: fibrinogen, ESR, and platelet count. These are prothrombotic parameters that demonstrate the body's response to tissue injury following trauma. However, there is still inadequate evidence to conclude that these three biomarkers can predict VTE related to a hypercoagulable state post-trauma. Further research is needed to continue similar studies with a larger sample size and a longer study period. These three biomarkers, on the other hand, could be used as additional parameters to support prophylaxis indications against VTE in patients with high-risk trauma that require immobilization.
